# Impact of percutaneous coronary intervention on biomarker levels in patients in the subacute phase following myocardial infarction: the Occluded Artery Trial (OAT) biomarker ancillary study

**DOI:** 10.1186/1471-2261-13-91

**Published:** 2013-10-24

**Authors:** Mariusz Kruk, Venu Menon, Jacek Kądziela, Zygmunt Sadowski, Witold Rużyłło, Jadwiga Janas, Marek Roik, Grzegorz Opolski, Krzysztof Zmudka, Piotr Czunko, Michal Kurowski, Benita Busz-Papież, Elzbieta Zinka, Wojciech Jablonski, Krystyna Jaworska, Anna Raczynska, Grzegorz Skonieczny, Sandra Forman, Daner Li, Judith Hochman

**Affiliations:** 1 Institute of Cardiology, Warsaw, Poland; 2 Cleveland Clinic, Cleveland, Ohio, USA; 3 Medical University, Warsaw, Poland; 4 John Paul II Hospital, Krakow, Poland; 5 County Hospital, Szczecin, Poland; 6 County Hospital, Koszalin, Poland; 7 County Hospital, Toruń, Poland; 8 Clinical Trials and Surveys Corporation, Owings Mills, MD, USA; 9 New York University School of Medicine, New York, New York, USA; 10 Cardiovascular Clinical Research Center, Leon Charney Division of Cardiology, New York University School of Medicine, 530 First Ave, Skirball 9R, New York, NY 10016, USA

**Keywords:** Acute coronary syndrome, Percutaneous coronary intervention, Biomarkers, Heart failure, Remodeling

## Abstract

**Background:**

The purpose of the Occluded Artery Trial (OAT) Biomarker substudy was to evaluate the impact of infarct related artery (IRA) revascularization on serial levels of *N*-*terminal* prohormone of brain natriuretic peptide (NT-proBNP) and dynamics of other biomarkers related to left ventricular remodeling, fibrosis and angiogenesis.

**Methods:**

Patients were eligible for OAT-Biomarker based on the main OAT criteria. Of 70 patients (age 60.8 ± 8.8, 25% women) enrolled in the substudy, 37 were randomized to percutaneous coronary intervention (PCI) and 33 to optimal medical therapy alone. Baseline serum samples were obtained prior to OAT randomization with follow up samples taken at one year. The primary outcome was percent change of NT-proBNP from baseline to 1 year. The secondary outcomes were respective changes of matrix metalloproteinases (MMP) 2 and 9, tissue inhibitor of matrix metalloproteinase 2 (TIMP-2), Vascular Endothelial Growth Factor (VEGF), and Galectin-3.

**Results:**

Paired (baseline and one-year) serum samples were obtained in 62 subjects. Baseline median NT-proBNP level was 944.8 (455.3, 1533) ng/L and decreased by 69% during follow-up (p < 0.0001). Baseline MMP-2 and TIMP-2 levels increased significantly from baseline to follow-up (p = 0.034, and p = 0.027 respectively), while MMP-9 level decreased from baseline (p = 0.038). Levels of VEGF and Galectin-3 remained stable at one year (p = NS for both). No impact of IRA revascularization on any biomarker dynamics were noted.

**Conclusions:**

There were significant changes in measured biomarkers related to LV remodeling, stress, and fibrosis following MI between 0 and 12 month. Establishing infarct vessel patency utilizing stenting 24 hours-28 days post MI did not however influence the biomarkers’ release.

## Background

Myocardial infarction (MI) initiates healing and repair responses, which include scar formation, angiogenesis and remodeling. Preliminary data suggested that late (beyond 24 hours) opening of occluded culprit infarct artery might have beneficial impact upon these processes, with subsequent clinical advantages [1]. The Occluded Artery Trial (OAT) was a randomized trial testing the clinical effect of mechanical reperfusion versus optimal medical therapy alone (MED) for totally occluded arteries in stable patients 3–28 calendar days following MI. The main trial was powered to evaluate clinical outcomes, and failed to show any additional benefit of the interventional treatment over optimal medical therapy [2]. The lack of observed clinical benefit with percutaneous coronary intervention (PCI) was also independent of the presence and degree of viability in the infarct zone [3]. Changing plasma levels of relevant biomarkers may reflect the dynamics of these processes more sensitively, and their assessment may provide additional pathophysiological insight into the main study clinical findings.

The primary purpose of the OAT Biomarker substudy was to evaluate the impact of mechanical revascularization on paired levels of *N*-*terminal* prohormone of brain natriuretic peptide (NT-proBNP) obtained at baseline and at 1 year of follow up; and secondly to assess the impact of the randomized treatment on dynamics of biomarkers related to post-infarction left ventricular remodeling, fibrosis and angiogenesis, including matrix metalloproteinases (MMP) 2 and 9 and tissue inhibitor of matrix metalloproteinase 2 (TIMP-2), Vascular Endothelial Growth Factor (VEGF), and galectin-3.

## Methods

### Study setting & population

The patients were eligible for OAT-Biomarker ancillary study when they consented for the parent trial OAT (ClinicalTrials.gov Identifier: NCT00004562). Due to lack of funding the study was performed in only 10 sites in Poland, two in Canada and a solitary site in the United States. The substudy was approved by Institutional Review Board at each participating center (Terenowa Komisja Bioetyczna Instytutu Kardiologii; University Health Network Research Ethics Board; Research Ethics Board, St. Michael’s Hospital; Ethics Committee, Truman Medical Center) and patients provided separate written informed consent for the OAT-Biomarker ancillary study. During follow-up repeated blood sampling 1 year after randomization was performed. For ninety percent (62/70) of the patients paired (baseline *plus* on-year) blood samples were available.

### Laboratory methods and study outcomes

Baseline samples were obtained prior to OAT randomization and repeated after one year. Blood samples were collected in tubes without anticoagulant. The samples were then centrifuged, and serum was stored frozen in aliquots at -20°C to -80°C at the enrolling site until shipped to the biomarker core laboratory at the Institute of Cardiology, where they were maintained at -80°C. Serum NT-proBNP (sandwich immunoassay) for this batched analysis were determined using an Elecsys 2010 (Roche Diagnostics GmbH, Mannheim, Germany). The analytic range of NT-proBNP assay extends from 5 to 35000 ng/L. The total coefficient of variation (CV) was 2.1% at a level of 181 ng/L and 1.8% at a level of 572 ng/L. Concentrations of matrix metalloproteinases, tissue inhibitor of metalloproteinase-2 and VEGF were assayed using quantitative sandwich enzyme immunoassay technique. The following Quantikine (R&D Systems, Inc., Minneapolis, MN, USA) tests were used: human/mouse MMP-2, total (sensitivity =0.16 ng/mL, intra- and inter-assay precision; CV = 5.7 and 8.2%), human MMP-9, total, (sensitivity = 0.156 ng/mL, intra- and inter-assay precision; CV = 2.9 and 7.9%), human TIMP-2 (sensitivity = 0.011 ng/mL, intra- and inter-assay precision; CV = 4.4 and 7.3%), human VEGF (sensitivity =9.0 pg/mL, intra- and inter-assay precision; CV = 4.5 and 7.0%). Concentration of galectin was estimated using human galectin-3 ELISA kit (Bender MedSystems GmbH, Vienna, Austria) (sensitivity = 0.12 ng/mL, intra- and inter-assay precision; CV = 6.4 and 11.4%).

The primary endpoint for this biomarker analysis was NT-proBNP percent change from baseline to 1 year. The dynamics of the other biomarkers comprised the secondary outcomes of the study. The primary clinical endpoint of OAT was a composite of death from any cause, reinfarction, or NYHA class IV heart failure with hospitalization or admission in a short-stay unit with follow up extending out to 7 years. The study clinical endpoints were adjudicated by an independent committee. The definition of reinfarction has been provided previously [2].

### Statistical analysis

Data management and statistical analyses were performed by the OAT data coordinating center. Baseline biomarkers were assessed as medians (interquartile range), clinical characteristics were assessed as frequencies/proportions or mean ± standard deviation (SD) and compared to the remaining main OAT patients, as well as by treatment groups (PCI vs. MED). The nonparametric median Wilcoxon test, t-test or chi square tests were used for comparisons as appropriate. The biomarker’s levels were presented as medians (inter-quartile range), and compared by means of Wilcoxon Signed Rank test. Two-sided two-group t-tests at alpha = 0.05 were done for comparisons of biomarkers changes from baseline to 1-year.

To examine the impact of the study treatment on biomarker dynamics, change in biomarkers’ levels between baseline and 1 year were compared by treatment assignment (PCI versus MED). The independent correlates of NT-proBNP and other biomarkers dynamics (change from baseline to one year levels) were assessed by means of regression analyses. The univariate models were run first to identify any predictor significant at 0.10 level. Then a backward selection was run on the multivariate model to determine the final predictors that were still significant at 0.05 level. The treatment group was forced into the multivariable models. For the biomarkers dynamics (baseline *versus* follow-up), biomarkers dynamics between PCI *versus* MED subgroups, and for regression models with NT-proBNP dynamics as the dependent variable, significance level was set at p < 0.05. For other analyses significance level was set at p < 0.01. All analyses are performed on an intention-to-treat basis.

With a targeted sample size of 200 patients and a predicted standard deviation of 50% for change in biomarker levels from baseline to 1 year, there would be 80% power to detect a 22% difference in NT-proBNP change between the treatment groups in the presence of a 15% drop-out rate. With the observed SD of 76% and sample size of 62 (paired samples), there was 80% power to detect a difference of 56% for the primary outcome of change in NT-proBNP levels from baseline to 1 year.

## Results

### Baseline characteristics

Patient characteristics at study baseline and comparison to the main OAT cohort are illustrated in Table 1. The mean age of enrolled patients was 60.8 ± 8.8 years, all patients were white (p < 0.001 when compared to the main OAT), and 17 (25%) were women. Patients enrolled in the biomarker substudy had a longer time from MI to randomization, less thrombolytic therapy for the index event, lower prevalence of multivessel disease, and higher use of lipid lowering medications and spironolactone (Table 1). Overall, thirty seven biomarker study subjects were randomized to PCI while 33 were in the control arm. Patients randomized to medical therapy alone had less diabetes and received less thienopyridines, when compared to the PCI group (Table 2).

**Table 1:** Baseline characteristics

	***OAT-Biomarker (N = 70)***		***Remaining OAT (N = 2131)***		
	***n***	***% (mean ± sd)***	***N***	***% (mean ± sd)***	***p-value***
Age (years)	70	(60.8 ± 8.8)	2131	(58.5 ± 11)	0.097
Women	17	24.3	467	21.9	0.637
Race White	70	100	1693	79.4	<.001
**Clinical history**					
Angina	15	21.4	480	22.5	0.829
Myocardial Infarction	3	4.3	244	11.5	0.080
Percutaneous intervention	4	5.7	101	4.7	0.574
Congestive heart failure	1	1.4	51	2.4	1.000
Hypertension	36	51.4	1035	48.6	0.638
Diabetes	10	14.3	444	20.8	0.183
Insulin use	6	8.6	118	5.5	0.279
Hypercholesterolemia	31	44.3	1111	52.2	0.195
Stroke	4	5.7	59	2.8	0.138
Cerebrovascular disease	5	7.1	77	3.6	0.125
Peripheral vascular disease	1	1.4	82	3.9	0.519
Current Smoker	25	35.7	834	39.1	0.564
Family history CAD	29	41.4	854	40.1	0.820
Days from MI to randomization - median {IQR}	70	21{16–26}	2131	8{5–16}	<.001
**Index myocardial infarction**					
Received thrombolytics	1	1.4	423	19.9	<.001
STE / Q wave / R loss	58	82.9	1849	86.8	0.344
Killip Class II-IV	11	15.7	406	19.1	0.473
Ejection Fraction	70	(46.3 ± 9.7)	2115	(47.8 ± 11.1)	0.275
Rales	1	1.4	136	6.4	0.125
**Clinical measures**					
Estimated GFR <60 ml/min/1.73 m^2^	8	11.4	305	14.6	0.453
Glucose > =126 mg/dl	8	11.6	556	28.8	0.002
BMI (kg/m^2^)	69	(26.7 ± 3.6)	2118	(28.6 ± 5.1)	0.003
Heart rate (bpm)	70	(69.3 ± 9.8)	2128	(71.9 ± 12)	0.076
Systolic blood pressure (mmHg)	70	(120.4 ± 11.9)	2129	(120.8 ± 18.1)	0.831
**Angiography**					
Infarct-related artery (IRA) –LAD	19	27.1	774	36.3	. 0.267
Infarct-related artery (IRA) –Lcx	11	15.7	324	15.2	
Infarct-related artery (IRA) –RCA	40	57.1	1033	48.5	
Multivessel Disease	21	30.4	358	16.9	0.004
Collaterals	64	92.8	1858	88.3	0.256
**Medications**					
Aspirin	68	97.1	2037	95.6	0.767
Thienopyridine	42	60	1287	60.4	0.947
Warfarin	3	4.3	212	9.9	0.150
Beta blocker	65	92.9	1867	87.6	0.187
Calcium blocker	2	2.9	127	6	0.434
Lipid lowering agent	68	97.1	1720	80.7	<.001
Spironolactone	9	12.9	115	5.4	0.008
ACE-Inhibitor or ARB	62	88.6	1709	80.2	0.082

ACE-angiotensin converting enzyme, ARB-angiotensin receptor blocker, BMI-body mass index, BP, blood pressure, CAD-coronary artery disease, GFR-glomerular filtration rate, IQR-interquartile ranges, IRA-infarct related artery, LAD-left anterior descending artery, LCX-left circumflex coronary artery, RCA-right coronary artery, STE-ST elevation.

**Table 2:** Baseline biomarker and patient characteristics by treatment assignment

	***PCI (N = 37)***		***MED (N = 33)***		
	***n***	% (mean ± sd) median{interquartile range}*	***n***	% (mean ± sd) median{interquartile range}*	***p-value***
**Biomarkers**					
Baseline NT-proBNP(ng/L){IQR}	37	846.1{402.4,1488}	33	1100{474.6,1726}	0.306
Baseline VEGF(pg/mL){IQR}	31	42.5{23.6,207.2}	27	37.5{17.4,70.2}	0.293
Baseline MMP-2(ng/mL){IQR}	33	185.7{172.3,216.9}	28	185.3{172.1,239.7}	0.879
Baseline MMP-9(ng/mL){IQR}	33	90{56,185}	28	102.3{44.2,163.2}	0.506
Baseline TIMP-2(ng/mL){IQR}	33	64.5{57.6,74.1}	28	64.3{59.2,70}	0.789
Baseline GALECTIN(ng/L){IQR}	33	12.8{9.8,15.5}	28	13{9.2,14.9}	0.806
Age (years)	37	(62.5 ± 9.1)	33	(58.8 ± 8.1)	0.086
Female	11	29.7	6	18.2	0.261
Race White	37	100	33	100	.
**Clinical History**					
Angina	8	21.6	7	21.2	0.967
Myocardial infarction	1	2.7	2	6.1	0.599
Percutaneous coronary intervention	3	8.1	1	3	0.616
Congestive heart failure	0	0	1	3	0.471
Hypertension	21	56.8	15	45.5	0.345
Diabetes	2	5.4	8	24.2	0.038
Insulin use	1	2.7	5	15.2	0.093
Hypercholesterolemia	15	40.5	16	48.5	0.504
Stroke	2	5.4	2	6.1	1.000
Cerebrovascular disease	2	5.4	3	9.1	0.661
Peripheral vascular disease	1	2.7	0	0	1.000
Current Smoker	13	35.1	12	36.4	0.915
Family history CAD	12	32.4	17	51.5	0.106
Days from MI to randomization - median {IQR}	37	22{17–26}	33	20{14–25}	0.135
**Index myocardial infarction**					
Received thrombolytics	1	2.7	0	0	1.000
STE / Q wave / R loss	30	81.1	28	84.8	0.676
Killip Class II-IV	7	18.9	4	12.1	0.522
Ejection fraction	37	(46.8 ± 9)	33	(45.7 ± 10.5)	0.652
Rales	0	0	1	3.1	0.464
**Clinical measures**					
Estimated GFR <60 ml/min/1.73 m^2^	5	13.5	3	9.1	0.714
Glucose > =126 mg/dl	2	5.6	6	18.2	0.140
BMI (kg/m^2^)	37	(26.4 ± 2.8)	32	(27.2 ± 4.3)	0.365
Heart rate (bpm)	37	(68.9 ± 9.4)	33	(69.7 ± 10.3)	0.733
Systolic blood pressure (mmHg)	37	(119.5 ± 11.4)	33	(121.4 ± 12.5)	0.507
**Angiography**					
Infarct-related artery (IRA) LAD	13	35.1	6	18.2	0.240
Infarct-related artery (IRA) Lcx	6	16.2	5	15.2	
Infarct-related artery (IRA) RCA	18	48.6	22	66.7	
Angiography - multivessel disease	9	24.3	12	37.5	0.236
Collaterals	36	97.3	28	87.5	0.175
**Medications**					
Aspirin	36	97.3	32	97	1.000
Thienopyridine	34	91.9	8	24.2	<.001
Warfarin	0	0	3	9.1	0.100
Beta blocker	32	86.5	33	100	0.056
Ca blocker	1	2.7	1	3	1.000
Lipid lowering agent	35	94.6	33	100	0.494
Spironolactone	6	16.2	3	9.1	0.485
ACE-Inhibitor or ARB	32	86.5	30	90.9	0.714

*median(interquartile range) for biomarkers, ACE-angiotensin converting enzyme, ARB-angiotensin receptor blocker, BMI-body mass index, BP = blood pressure, CAD-coronary artery disease, GFR-glomerular filtration rate, IQR-interquartile ranges, IRA-infarct related artery, LAD-left anterior descending artery, LCX-left circumflex coronary artery, MED – medical therapy alone, PCI- percutaneous coronary intervention, RCA-right coronary artery, STE-ST elevation.

### Biomarkers

**Figure 1:**
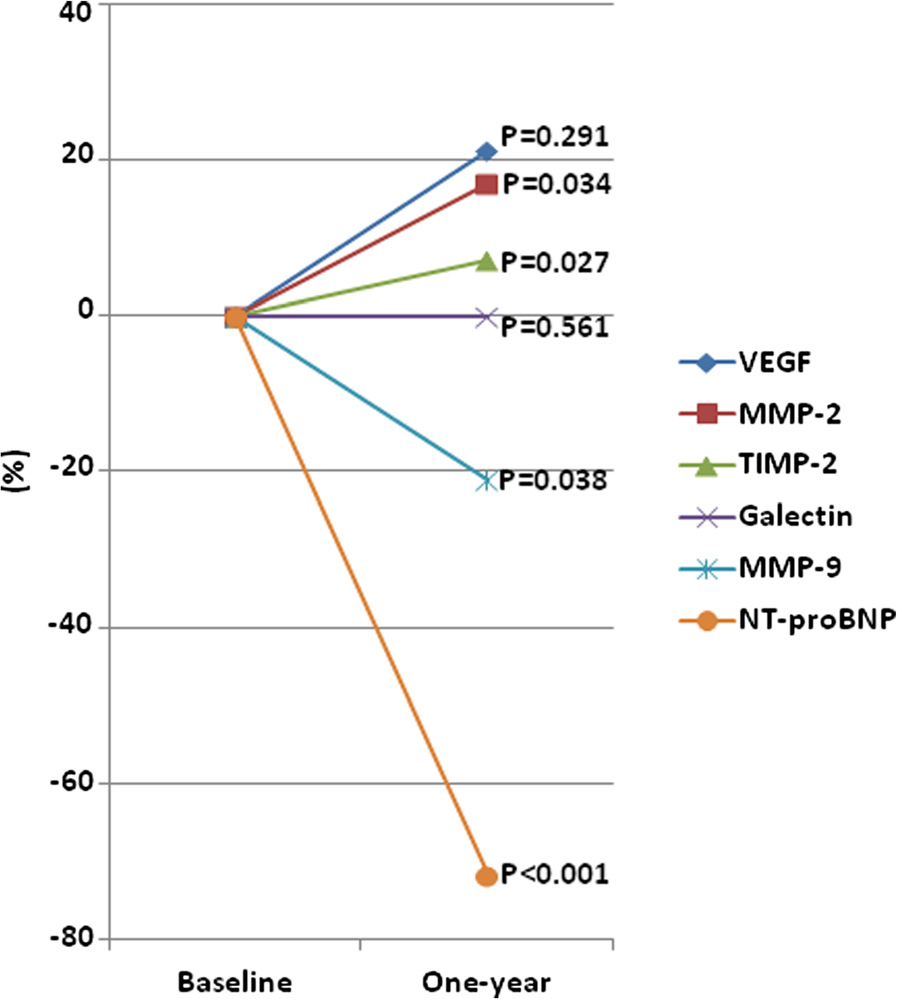
**Baseline to one-year, median percent change of the biomarker levels; NT-proBNP- *N*-*terminal *** **prohormone of brain natriuretic peptide; MMP-matrix metalloproteinase; TIMP - tissue inhibitor of matrix metalloproteinase; VEGF-Vascular Endothelial Growth Factor.**

Baseline levels of NT-proBNP were 944.8 (455.3, 1533) ng/L and decreased by 69% to 266.8 (138.2, 584.9) ng/L during follow-up (p < 0.0001). During this period MMP-2 [185.7(172.3, 219.4) ng/mL versus 216.9 (176, 249.9) ng/mL, p = 0.034] and TIMP-2 levels [64.5 (58.6, 71) ng/mL versus 69.1 (62.6, 74.4) ng/mL, p = 0.027] increased significantly from baseline to follow-up. In contrast, MMP-9 levels decreased from 101.6 (53.5, 170.2) ng/mL to 80.2 (43.7, 150.2) ng/mL (p = 0.038) while levels of VEGF [39.9 (21.7, 83.7) pg/mL vs. 48.3 (19.6, 82.5) p = 0.291], and Galectin [12.8 (9.4, 15.2) ng/mL vs. 12.8 (10.1, 15.4) ng/mL, p = 0.561] remained stable (Figure 1).

### Impact of PCI on biomarker levels

**Figure 2:**
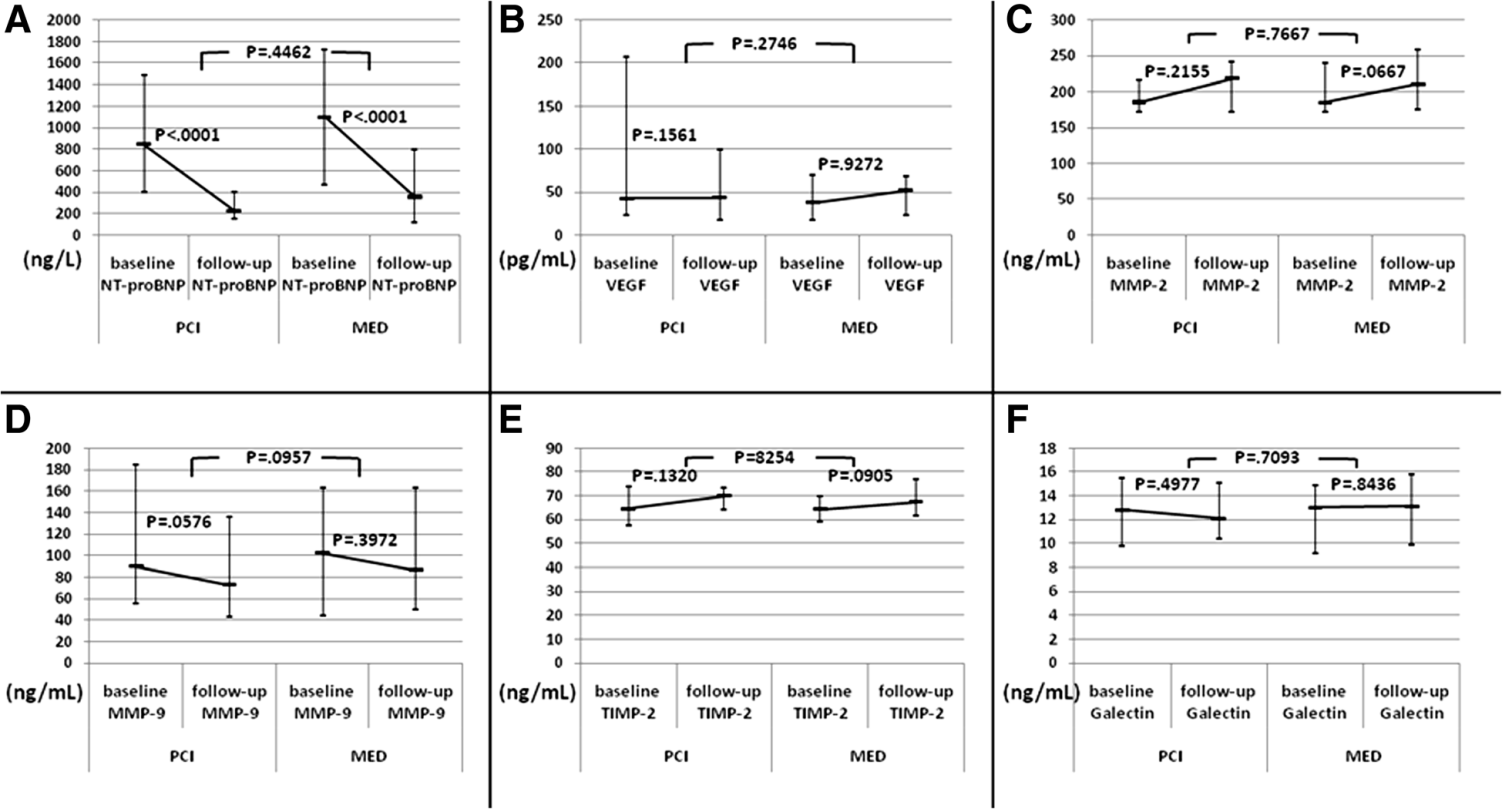
**Baseline to one-year changes of the examined biomarkers’ levels by the study treatment (recanalization of infarct related artery (PCI) versus medical therapy alone (MED)). (A)** - NT-proBNP- *N*-*terminal* prohormone of brain natriuretic peptide; **(B)** - VEGF-Vascular Endothelial Growth Factor; **(C)** - MMP-2-matrix metalloproteinase-2; **(D)** - MMP-9-matrix metalloproteinase-9; **(E)** - TIMP-2 - tissue inhibitor of matrix metalloproteinase-2; **(F)** - Galectin.

The percent change from baseline to one year levels of NT-proBNP was significant within both PCI and MED subgroups. There was a trend toward increased MMP-2 in medically treated patients only, and MMP-9 tended to decrease in PCI but not in medically treated patients (Figure 2). Follow-up levels of the examined biomarkers and their changes over time did not differ significantly for the PCI versus MED subgroups (Figure 2).

### Predictors of the biomarkers’ dynamics

The univariate and multivariable predictors of biomarker changes over time are provided in Table 3. Percent change of NT pro-BNP was predicted independently by its baseline level and family history of coronary disease. For the other biomarkers, their dynamics were independently predicted by their respective baseline values. Additionally, changes in VEGF were correlated to previous thrombolytic therapy; while changes in TIMP-2 were related to history of renal insufficiency, baseline heart rate and BMI. Galectin changes were related to BMI (Table 3).

**Table 3:** Uni- and multivariable predictors of the biomarkers dynamics (change from baseline to one-year)

		**Univariable model**		**Multivariable model**	
		**Estimate**	**ProbChiSq**	**Estimate**	**ProbChiSq**
**NT-proBNP**	Baseline NT-proBNP	**-0.699**	**<.001**	**-0.695**	**<.001**
	PCI versus med	**-6.939**	**0.979**	**-253.604**	**0.162**
	Famliy history of CAD	**-511.007**	**0.052**	**-448.534**	**0.015**
	New Q waves	**-531.444**	**0.067**	**-**	**-**
**VEGF**	Baseline VEGF	**-0.750**	**<.001**	**-0.727**	**<.001**
	PCI versus med	**-42.952**	**0.109**	**-5.514**	**0.694**
	History of cerebrovascular disease	**-108.966**	**0.065**	**-**	**-**
	Current smoker	**-53.395**	**0.049**	**-**	**-**
	Thrombolytic therapy	**220.645**	**0.026**	**171.550**	**<0.001**
**MMP-2**	Baseline MMP-2	**-0.982**	**<.001**	**-0.979**	**<.001**
	PCI versus med.	**-51.316**	**0.351**	**-11.677**	**0.384**
	systolic blood pressure (10 mmHg)	**40.239**	**0.079**	**-**	**-**
	diastolic blood pressure (10 mmHg)	**62.034**	**0.053**	**-**	**-**
**MMP-9**	Baseline MMP-9	**-0.751**	**<.001**	**-0.742**	**<.001**
	PCI versus med.	**-24.601**	**0.436**	**0.526**	**0.981**
	MI history	**122.755**	**0.081**	**104.471**	**0.032**
	History of cerebrovascular disease	**-185.871**	**0.006**	**-**	**-**
	Current smoker	**-58.054**	**0.068**	**-**	**-**
**TIMP-2**	Baseline TIMP-2	**-0.919**	**<.001**	**-0.885**	**<.001**
	PCI versus med.	**0.412**	**0.914**	**-2.558**	**0.255**
	Male versus Female	**8.349**	**0.049**	**-**	**-**
	History of Renal Insufficiency	**17.714**	**0.085**	**22.685**	**0.003**
	Family history of CAD	**6.314**	**0.099**	**-**	**-**
	Current smoker	**-7.321**	**0.056**	**-5.025**	**0.029**
	Heart rate (10 beats/minute)	**3.213**	**0.099**	**3.566**	**0.002**
	BMI (10 kg/m^2^)	**-9.403**	**0.087**	**-11.541**	**<0.001**
**GALECTIN**	Baseline GALECTIN	**-0.82**	**<.001**	**-0.77**	**<.001**
	PCI versus med.	**-0.734**	**0.674**	**0.037**	**0.975**
	Angina history	**-3.418**	**0.087**	**-**	**-**
	PCI history	**6.348**	**0.059**	**-**	**-**
	Thrombolytic therapy	**-13.353**	**0.040**	**-10.305**	**0.023**
	BMI (10 kg/m^2^)	**8.281**	**<0.001**	**7.326**	**<.001**
	Ejection fraction (10%)	**-1.983**	**0.018**	**-**	**-**

NT-proBNP- *N*-*terminal *prohormone of brain natriuretic peptide, matrix metalloproteinase-MMP, tissue inhibitor of matrix metalloproteinases- TIMP, Vascular Endothelial Growth Factor -VEGF.

BMI-body mass index, CAD-coronary artery disease, MED-medical therapy, MMP-matrix metalloproteinase; NT-proBNP- *N*-*terminal *prohormone of brain natriuretic peptide; PCI-percutaneous coronary intervention; TIMP - tissue inhibitor of matrix metalloproteinase; VEGF-Vascular Endothelial Growth Factor.

## Discussion

This is a small, serial measurement of biomarkers substudy performed under the auspices of a large phase 3 rigorously performed randomized clinical trial. Despite its small size, the unique patient characteristics and the protocol assigned PCI in the background of a late totally occluded infarct related artery makes these observations notable. The differences in baseline characteristics from the parent trial likely reflect geographic characteristics arising from randomization in Poland which accounted for all of the paired samples considered for analysis. Prior analyses of the main trial demonstrated similar treatment effect across regions. Our study demonstrated that the plasma levels of biomarkers related to LV function and remodeling, including NT-proBNP, MMP-2, TIMP-2 and MMP-9, significantly evolved from baseline to one year following index MI. However, the change in biomarkers levels were not significantly modified by mechanical opening of the occluded infarct related artery (IRA) performed 3 to 28 days post MI.

NT-proBNP is an established biomarker used for diagnosis, prognosis and in selected cases management of patients with heart failure or acute coronary syndromes. It is synthesized in the ventricular myocardium in response to increased wall tension, myocardial stretch or ischemia [4-6]. The kinetics of NT-proBNP in the early post infarction period have been previously reported. An early rise is seen beginning immediately after the index infarct that has a second peak at about 24 hours. Subsequently NT-proBNP levels plateau at approximately 35 days. Plasma levels of NTpro-BNP chave been shown to correlate with cardiac function following myocardial infarction [7], as well as with mortality in heart failure or acute coronary syndromes. Serial changes of NT-proBNP parallel improving or worsening LV function [8,9]. In a substudy of FRISC-II trial, higher baseline levels of NT-proBNP were shown to identify the subgroup of NSTE-ACS patients benefitting from interventional treatment [10]. The decline of NT-proBNP observed in OAT-Biomarker likely reflects gradual improvement of cardiac function (ejection fraction) directly observed in OAT-NUC and TOSCA 2 ancillary studies [3,11]. We observed a lack of impact of the IRA recanalization on biomarker changes over one year. These findings are consistent with the lack of clinical benefit observed with PCI in the parent trial. Importantly, majority (89%) of the study patients received ACE inhibitors or angiotensin receptor-1 blockers, therefore the hypothetical impact of the study PCI was tested on top of optimal medical therapy, which itself had been shown to lower natriuretic peptide levels in patients with LV dysfunction [12].

Matrix metalloproteinases (MMP) and their tissue inhibitors (TIMPs) are enzymes involved in the extracellular matrix turnover and cardiac remodeling following infarction. Their release and roles following MI are time dependent [13,14]. Prior studies reported a cause-effect relation between MMP-2,-9 and adverse LV myocardial remodeling after MI [15,16]. MMP-2 and -9 levels were shown to correlate with echo measures of LV remodeling and non scarred myocardial mass on CMR [17-19]. Existing evidence supports clinical testing of MMP inhibitors for prevention of postinfarction remodeling in humans [13,20].

In our cohort the values of MMP-2 significantly increased, while MMP-9 decreased from baseline. Similar shifts in these biomarker levels during long-term follow-up were previously observed in patients after MI [17,18,21,22]. In our study, PCI tended to decrease MMP-9 levels more significantly than medical therapy alone (respectively 38% vs. 5%). Regarding MMP-2, PCI had no visible effect on its dynamics.

TIMP-2 may either inhibit or stimulate activation of MMP-2 [23,24]. Its deficiency has been shown to accelerate post infarction LV remodeling in an animal model [25]. Plasma levels of TIMP-2 have been shown to increase in late stages of MI, as we observed in our cohort [17]. In the current study, TIMP-2 rise was positively correlated with history of renal insufficiency and heart rate and negatively with body mass index. However, PCI of IRA did not significantly impact alterations in these marker dynamics.

VEGF is implicated in neovascularization of ischemic tissue and wound healing, and its levels were previously shown to be correlated with peak creatine kinase levels in patients with MI. VEGF elevation has also been shown to be associated with clinical myocardial ischemia and adverse outcomes following ACS [26,27]. The only independent correlate of the VEGF dynamics in our study was thrombolytic therapy for the index event. We did not find any association between the study treatment and baseline to one-year change of VEGF levels.

Experimental observations suggest that galectin-3 may be regarded not only as a biomarker but also as an active mediator of fibrosis causing progression of heart failure [28,29]. In the clinical setting galectin-3 levels have been shown to correlate with clinical outcomes in patients with heart failure, providing incremental prognostic value to NT-proBNP [30]. In our study no significant difference was found between median baseline and follow up values of galectin, nor between PCI and medically treated patients. However, multivariable analysis identified thrombolytic therapy and BMI as independent correlates of the biomarker dynamics over one-year.

### Limitations

The study cohort represents a small series of patients enrolled in the main OAT study. The power to detect small differences in the biomarker dynamics between the PCI and MED groups was limited due to the insufficient recruitment relative to initial projections. It is also conceivable that the differences between the study groups would differ had the patients been followed for a longer period of time. Only paired samples at baseline and 1 year of follow up were available. As a result the time course of changes during this window could not be elicited.

## Conclusions

The Biomarker ancillary study to OAT suggests that there are significant dynamic changes (from baseline to one year) in biomarkers related to LV remodeling, stress, and fibrosis following MI, however, their dynamics are not significantly modified by opening of occluded IRA 3 days-28 days post MI. Consistent with the findings of the parent trial and the nuclear substudy, our findings appear to also suggest that late IRA recanalization does not significantly impact mechanisms underlying LV healing/remodeling processes following MI in patients on optimal medical therapy.

## Competing interests

The authors declare that they have no competing interests.

## Authors’ contributions

MK – 1) have made substantial contributions to conception and design, acquisition of data, analysis and interpretation of data; 2) have been involved in drafting the manuscript; and 3) have given final approval of the version to be published; VM, ZS, WR, JH, GO, KZ, SF, DL have made substantial contributions to conception and design, analysis and interpretation of data; 2) have been involved in revising the manuscript critically for important intellectual content; and 3) have given final approval of the version to be published; JK, JJ, MR, PC, MK, BBP, EZ, WJ, KJ, AR, GS 1) have made substantial contributions to acquisition of data; 2) have been involved in revising the manuscript critically for important intellectual content; and 3) have given final approval of the version to be published.

## Pre-publication history

The pre-publication history for this paper can be accessed here:

http://www.biomedcentral.com/1471-2261/13/91/prepub

## References

[B1] Sadanandan S Buller C Menon V Dzavik V Terrin M Thompson B Lamas G Hochman JS The late open artery hypothesis–a decade later Am Heart J 2001 142 411 421 10.1067/mhj.2001.117774 11526353

[B2] Hochman JS Lamas GA Buller CE Coronary intervention for persistent occlusion after myocardial infarction N Engl J Med 2006 355 2395 2407 10.1056/NEJMoa066139 17105759 PMC1995554

[B3] Udelson JE Pearte CA Kimmelstiel CD The Occluded Artery Trial (OAT) Viability Ancillary Study (OAT-NUC): influence of infarct zone viability on left ventricular remodeling after percutaneous coronary intervention versus optimal medical therapy alone Am Heart J 2011 161 611 621 10.1016/j.ahj.2010.11.020 21392619 PMC3073850

[B4] Levin ER Gardner DG Samson WK Natriuretic peptides N Engl J Med 1998 339 321 328 10.1056/NEJM199807303390507 9682046

[B5] Goetze JP Christoffersen C Perko M Increased cardiac BNP expression associated with myocardial ischemia FASEB J 2003 17 1105 1107 12709407 10.1096/fj.02-0796fje

[B6] Goetze JP Gore A Møller CH Steinbrüchel DA Rehfeld JF Nielsen LB Acute myocardial hypoxia increases BNP gene expression FASEB J 2004 18 1928 1930 15576492 10.1096/fj.03-1336fje

[B7] Richards AM Nicholls MG Yandle TG Plasma N-terminal pro-brain natriuretic peptide and adrenomedullin: new neurohormonal predictors of left ventricular function and prognosis after myocardial infarction Circulation 1998 97 1921 1929 10.1161/01.CIR.97.19.1921 9609085

[B8] Gackowski A Isnard R Golmard JL Comparison of echocardiography and plasma B-type natriuretic peptide for monitoring the response to treatment in acute heart failure Eur Heart J 2004 25 1788 1796 10.1016/j.ehj.2004.07.038 15474693

[B9] Michtalik HJ Yeh HC Campbell CY Acute changes in N-terminal pro-B-type natriuretic peptide during hospitalization and risk of readmission and mortality in patients with heart failure Am J Cardiol 2011 107 1191 1195 10.1016/j.amjcard.2010.12.018 21296322

[B10] Jernberg T Lindahl B Siegbahn A N-terminal pro-brain natriuretic peptide in relation to inflammation, myocardial necrosis, and the effect of an invasive strategy in unstable coronary artery disease J Am Coll Cardiol 2003 42 1909 1916 10.1016/j.jacc.2003.07.015 14662251

[B11] Dzavík V Buller CE Lamas GA Randomized trial of percutaneous coronary intervention for subacute infarct-related coronary artery occlusion to achieve long-term patency and improve ventricular function: the Total Occlusion Study of Canada (TOSCA)-2 trial Circulation 2006 114 2449 2457 10.1161/CIRCULATIONAHA.106.669432 17105848 PMC2785021

[B12] van Veldhuisen DJ Genth-Zotz S Brouwer J High- versus low-dose ACE inhibition in chronic heart failure: a double-blind, placebo-controlled study of imidapril J Am Coll Cardiol 1998 32 1811 1818 10.1016/S0735-1097(98)00464-1 9857856

[B13] Vanhoutte D Schellings M Pinto Y Heymans S Relevance of matrix metalloproteinases and their inhibitors after myocardial infarction: a temporal and spatial window Cardiovasc Res 2006 69 604 613 10.1016/j.cardiores.2005.10.002 16360129

[B14] Herzog E Gu A Kohmoto T Burkhoff D Hochman JS Early activation of metalloproteinases after experimental myocardial infarction occurs in infarct and non-infarct zones Cardiovasc Pathol 1998 7 307 312 10.1016/S1054-8807(98)00008-8 25851597

[B15] Peterson JT Hallak H Johnson L Matrix metalloproteinase inhibition attenuates left ventricular remodeling and dysfunction in a rat model of progressive heart failure Circulation 2001 103 2303 2309 10.1161/01.CIR.103.18.2303 11342481

[B16] Spinale FG Matrix metalloproteinases: regulation and dysregulation in the failing heart Circ Res 2002 90 520 530 10.1161/01.RES.0000013290.12884.A3 11909815

[B17] Webb CS Bonnema DD Ahmed SH Specific temporal profile of matrix metalloproteinase release occurs in patients after myocardial infarction: relation to left ventricular remodeling Circulation 2006 114 1020 1027 10.1161/CIRCULATIONAHA.105.600353 16923753

[B18] Orn S Manhenke C Squire IB Ng L Anand I Dickstein K Plasma MMP-2, MMP-9 and N-BNP in long-term survivors following complicated myocardial infarction: relation to cardiac magnetic resonance imaging measures of left ventricular structure and function J Cardiac Fail 2007 13 843 849 10.1016/j.cardfail.2007.07.006 18068618

[B19] Kelly D Cockerill G Ng LL Thompson M Khan S Samani NJ Squire IB Plasma matrix metalloproteinase-9 and left ventricular remodelling after acute myocardial infarction in man: a prospective cohort study Eur Heart J 2007 28 711 718 10.1093/eurheartj/ehm003 17339265 PMC2202923

[B20] Hudson MP Armstrong PW Ruzyllo W Effects of selective matrix metalloproteinase inhibitor (PG-116800) to prevent ventricular remodeling after myocardial infarction: results of the PREMIER (Prevention of Myocardial Infarction Early Remodeling) trial J Am Coll Cardiol 2006 48 15 20 10.1016/j.jacc.2006.02.055 16814643

[B21] Squire IB Evans J Ng LL Loftus IM Thompson MM Plasma MMP-9 and MMP-2 following acute myocardial infarction in man: correlation with echocardiographic and neurohumoral parameters of left ventricular dysfunction J Card Fail 2004 10 328 333 10.1016/j.cardfail.2003.11.003 15309700

[B22] Matsunaga T Abe N Kameda K Circulating level of gelatinase activity predicts ventricular remodeling in patients with acute myocardial infarction Int J Cardiol 2005 105 203 208 10.1016/j.ijcard.2005.01.011 16243114

[B23] Goldberg GI Strongin A Collier IE Genrich LT Marmer BL Interaction of 92-kDa type IV collagenase with the tissue inhibitor of metalloproteinases prevents dimerization, complex formation with interstitial collagenase, and activation of the proenzyme with stromelysin J Biol Chem 1992 267 4583 4591 1311314

[B24] Sato H Takino T Kinoshita T Cell surface binding and activation of gelatinase A induced by expression of membrane-type-1-matrix metalloproteinase (MT1-MMP) FEBS Lett 1996 385 238 240 10.1016/0014-5793(96)00389-4 8647259

[B25] Kandalam V Basu R Abraham T TIMP2 deficiency accelerates adverse post-myocardial infarction remodeling because of enhanced MT1-MMP activity despite lack of MMP2 activation Circ Res 2010 106 796 808 10.1161/CIRCRESAHA.109.209189 20056917

[B26] Hojo Y Ikeda U Zhu Y Expression of vascular endothelial growth factor in patients with acute myocardial infarction J Am Coll Cardiol 2000 35 968 973 10.1016/S0735-1097(99)00632-4 10732896

[B27] Heeschen C Dimmeler S Hamm CW Prognostic significance of angiogenic growth factor serum levels in patients with acute coronary syndromes Circulation 2003 107 524 530 10.1161/01.CIR.0000048183.37648.1A 12566361

[B28] de Boer RA Yu L van Veldhuisen DJ Galectin-3 in cardiac remodeling and heart failure Curr Heart Fail Rep 2010 7 1 8 10.1007/s11897-010-0004-x 20425490 PMC2831188

[B29] Sharma UC Pokharel S van Brakel TJ Galectin-3 marks activated macrophages in failure-prone hypertrophied hearts and contributes to cardiac dysfunction Circulation 2004 110 3121 3128 10.1161/01.CIR.0000147181.65298.4D 15520318

[B30] van Kimmenade RR Januzzi JL Jr Ellinor PT Utility of amino-terminal pro-brain natriuretic peptide, galectin-3, and apelin for the evaluation of patients with acute heart failure J Am Coll Cardiol 2006 48 1217 1224 10.1016/j.jacc.2006.03.061 16979009

